# Supplementary material for: Dissecting the heterogeneity of DENV vaccine-elicited cellular immunity using single-cell RNA sequencing and metabolic profiling
Source: Nat Commun. 2019 Aug 14;10:3666. doi: 10.1038/s41467-019-11634-7 (PMC6694189; doi:10.1038/s41467-019-11634-7)
Supplement: Supplementary file 1 — Supplementary Information [file 41467_2019_11634_MOESM1_ESM.pdf]

Supplementary Information for:

Dissecting the heterogeneity of DENV vaccine-elicited cellular immunity using single-cell RNA sequencing and metabolic profiling

Waickman et al

Supplementary Figure 1

A.

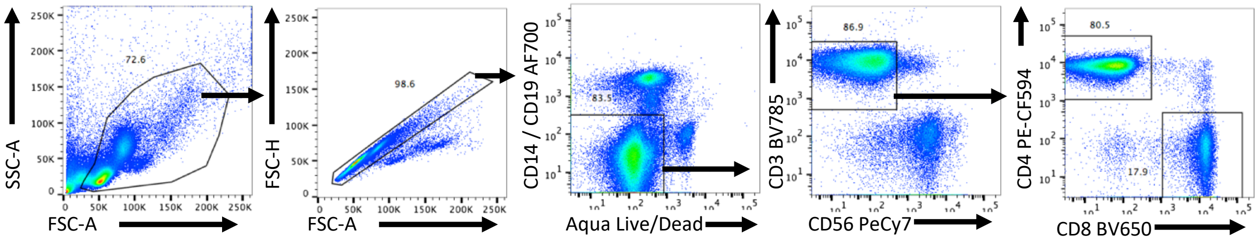

B.

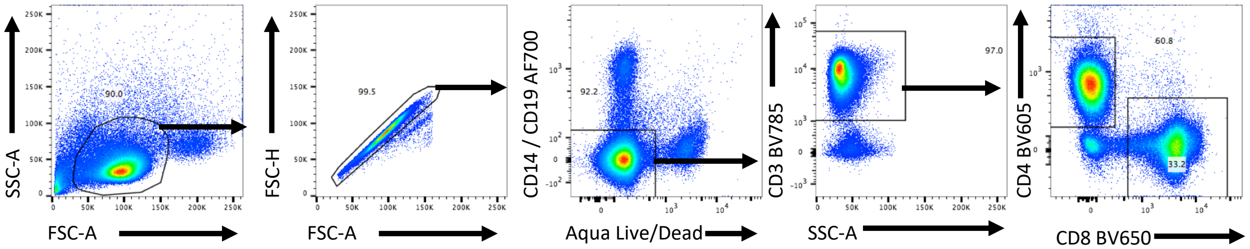

C.

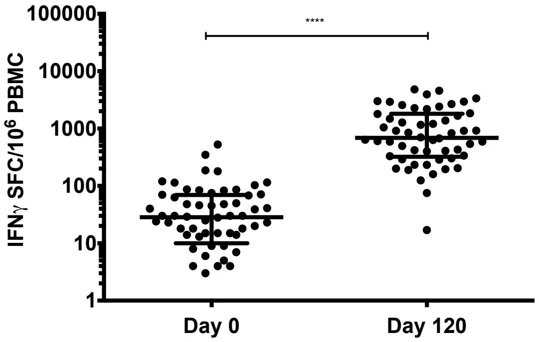

D.

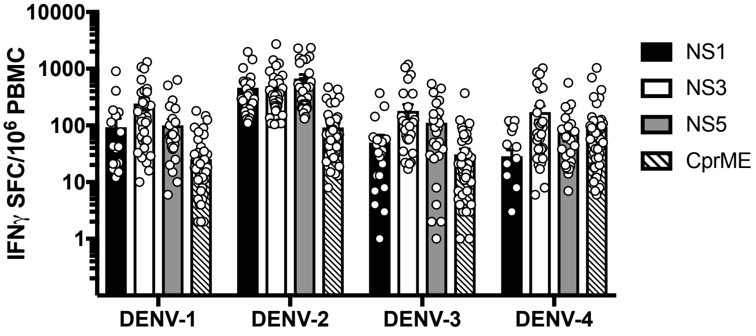

Supplementary Figure 1: A) Gating strategy for Figure 1A and Figure 1C. **B)** Gating strategy for Figure 1E. **C)** Quantification of total DENV-2 reactive IFN-g producing T cells from TAK-003-inoculated individuals on days 0 and 120 post vaccination as determined by ELISPOT. **D)** Antigen-specificity and serotype cross-reactivity of DENV-reactive, IFN-γ production T cells on day 120 post-vaccination as determined by ELISPOT. N = 55, *** p<0.0001 (Paired 2 tailed t-test). Source data are provided as a Source Data file

Supplementary Figure 2

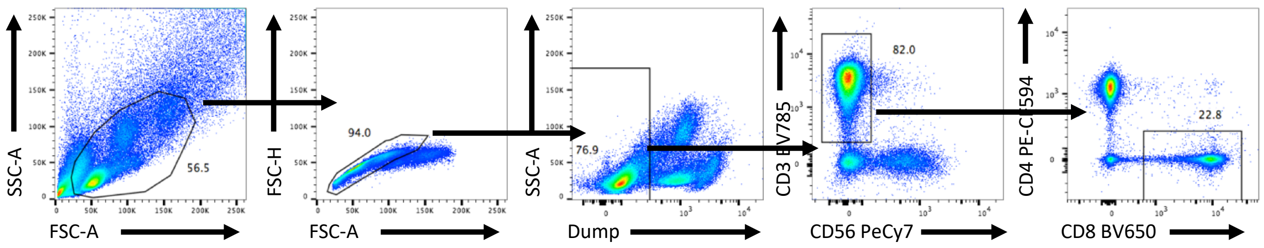

Supplementary Figure 2: Gating strategy for Figure 2B and Figure 2C. Dump channel contains viability marker (Aqua Live/Dead), CD14 BV510, and CD19 BV510.

Supplementary Figure 3

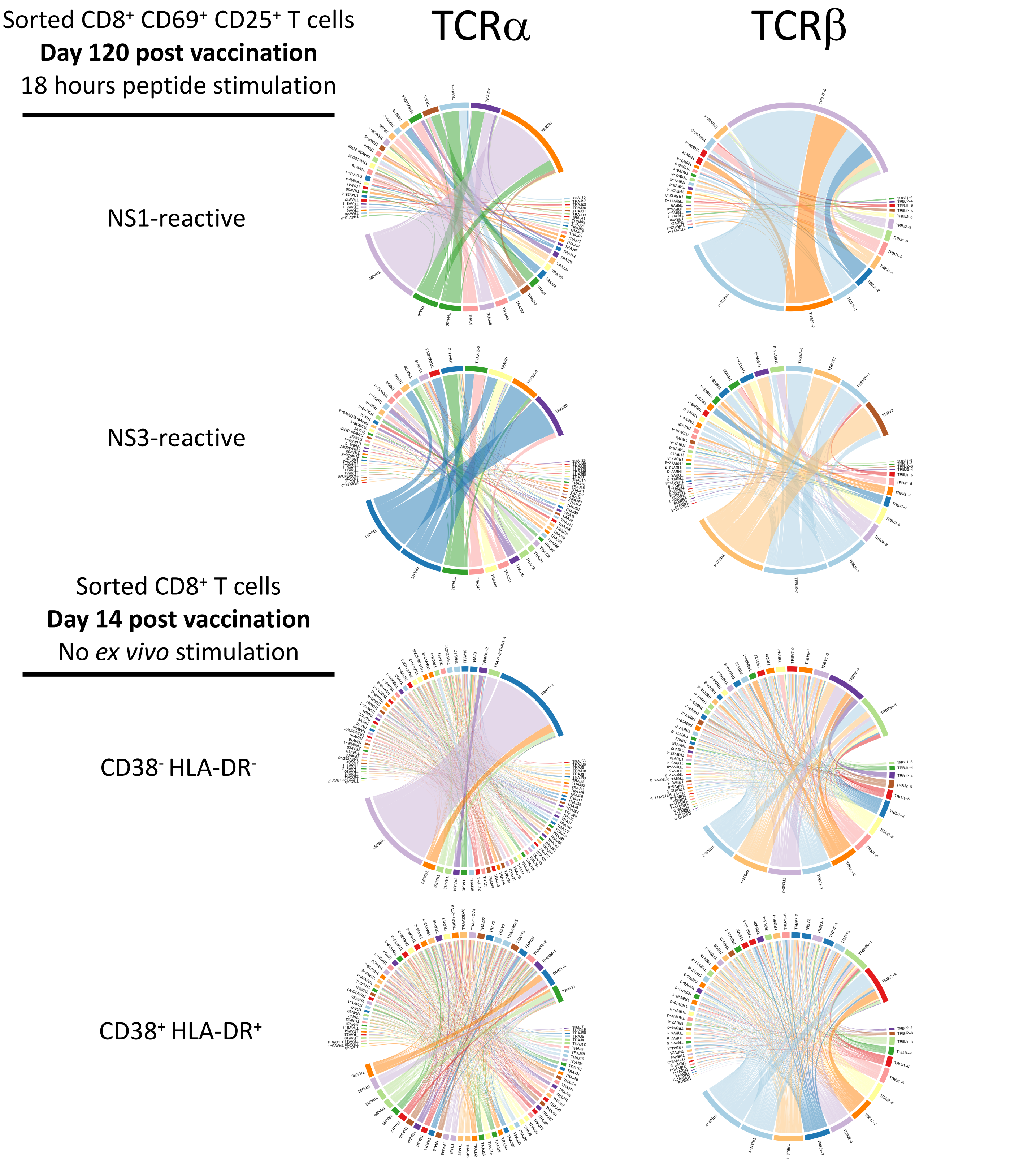

Supplementary Figure 3: A) TCRα and TCRβ V/J segment usage and pairing from sorted CD25⁺CD69⁺ CD8⁺ T cells isolated following *in vitro* stimulation with NS1- and NS3-derived peptide pools 120 days post TAK-003 administration. **B)** TCRα and TCRβ V/J segment usage and pairing from sorted CD38⁻HLA-DR⁻ (resting) CD8⁺ T cells and CD38⁺HLA-DR⁺ (activated) CD8⁺ T cells 14 days post TAK-003 administration

Supplementary Figure 4

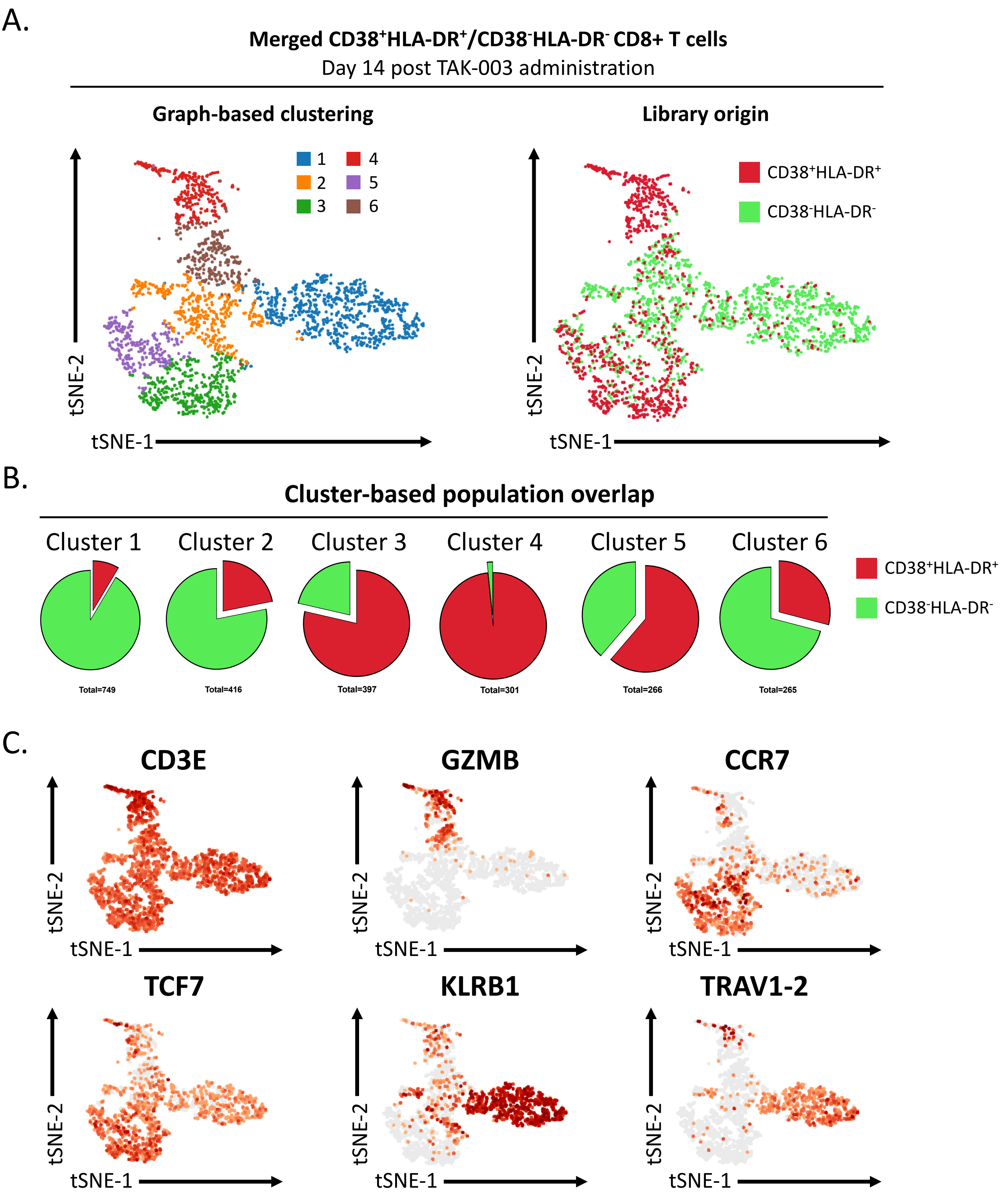

Supplementary Figure 4. scRNAseq analysis of sorted $CD38^{+}HLA\text{-}DR^{+}CD8^{+}$ T cells and sorted $CD38^{-}HLA\text{-}DR^{-}CD8^{+}$ T cells 14 days post TAK-003 administration. Gene expression datasets merged for downstream analysis

Supplementary Figure 5

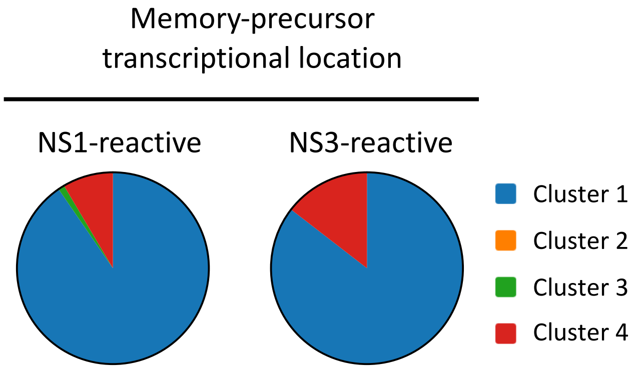

Supplementary Figure 5. Transcriptional cluster localization of NS1- and NS3- reactive CD8^{+} memory precursors at day 14 post-TAK-003 administration

Supplementary Figure 6

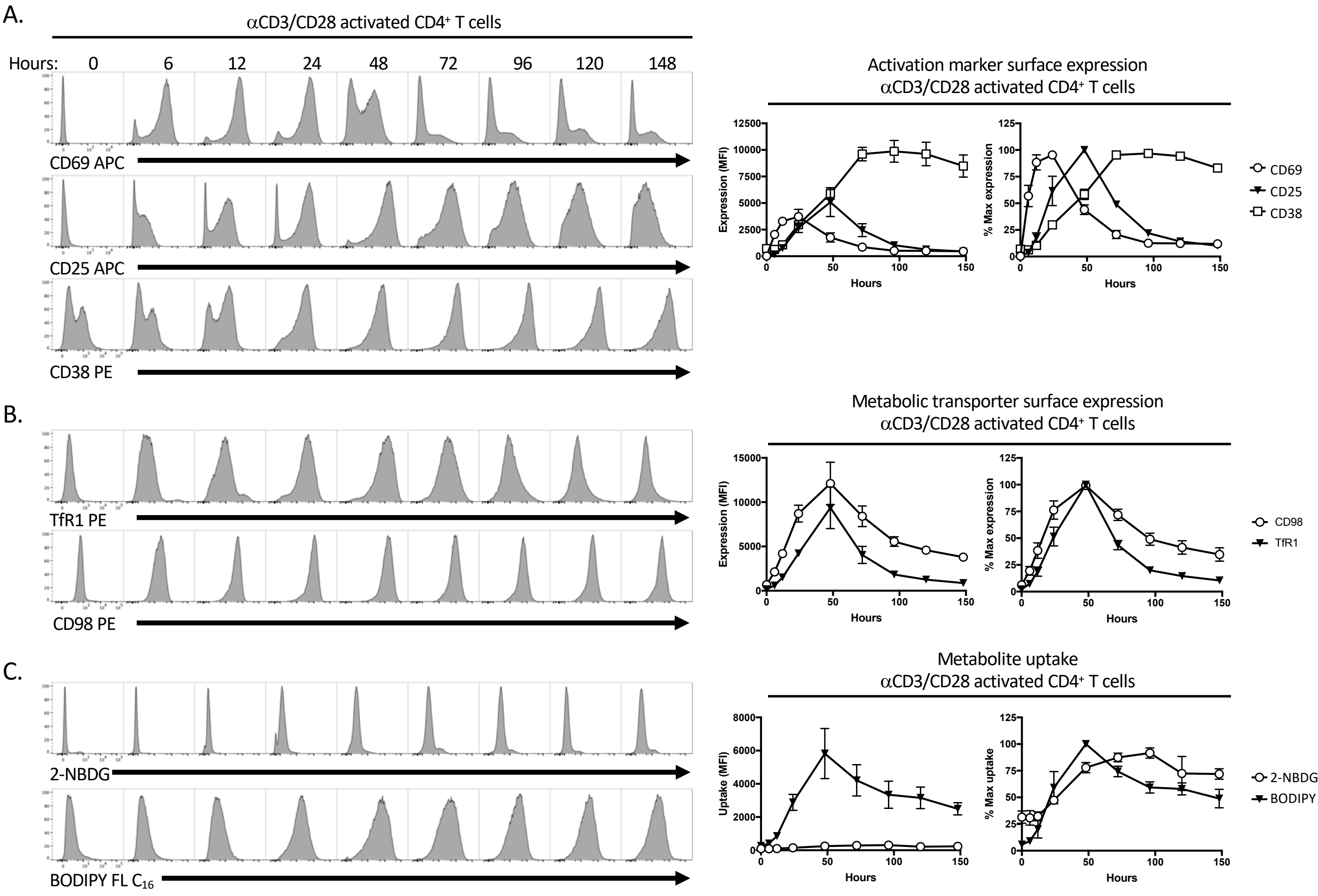

Supplementary Figure 6: Metabolic marker upregulation on *in vitro* stimulated CD4 T cells. PBMCs from healthy donors were stimulated with 0.1mg/ml aCD3 and 1mg/ml aCD28 and expression of **A)** CD69 and CD25 **B)** TfR1 and CD98, or **C)** uptake of 2-NBDG and BODIPY FL-C_{16} at the indicated timepoints. Results are representative of two independent experiments, with a total of 4 individual donors. Source data are provided as a Source Data file

Supplementary Figure 7

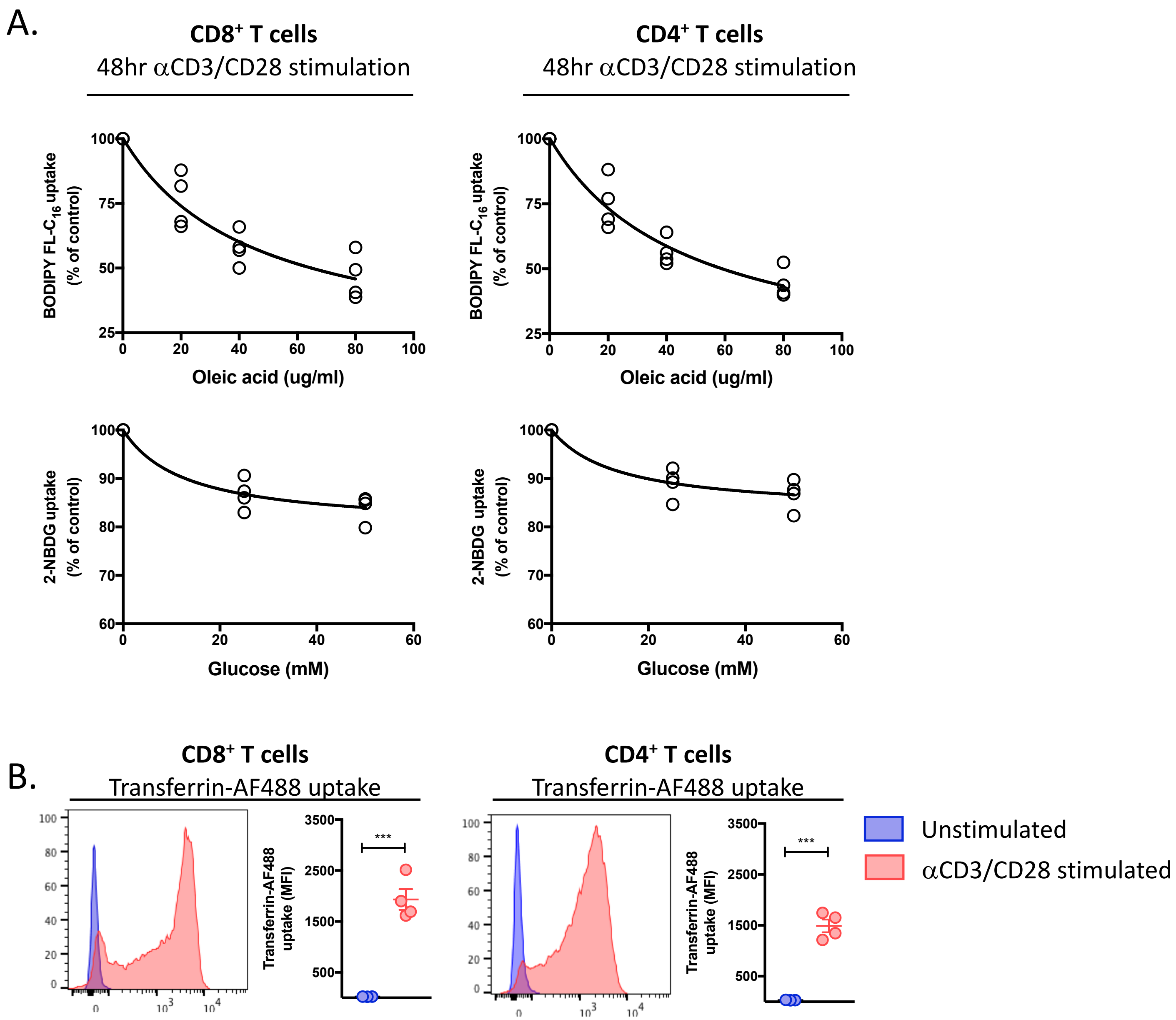

Supplementary Figure 7: A) Inhibition of BODIPY FL-C_{16} and 2-NBDG uptake in activated T cells by competition with unlabeled metabolites. PBMCs were stimulated with 0.1µg/mL of αCD3 and 1µg/ml αCD28 for 48hrs prior to analysis. BODIPY FL-C_{16} uptake was assessed in the presence or absence of 20-80µg/ml Oleic acid in complete cell culture media, while 2-NBDG uptake was assessed in the presence or absence of 25-50mM D-Glucose in glucose-free RPMI. Lines show non-linear fit analysis of inhibitor vs response (three parameters). **B)** Binding of Transferrin-AF488 by activated $CD8^+$ and $CD4^+$ T cells. PBMCs were stimulated with 0.1µg/mL of αCD3 and 1µg/ml αCD28 for 48hrs. Cells were washed in serum-free RPMI, then incubated for 30min with 50µg/mL of Transferrin-AF488, follow by surface staining. Source data are provided as a Source Data file

Supplementary Figure 8

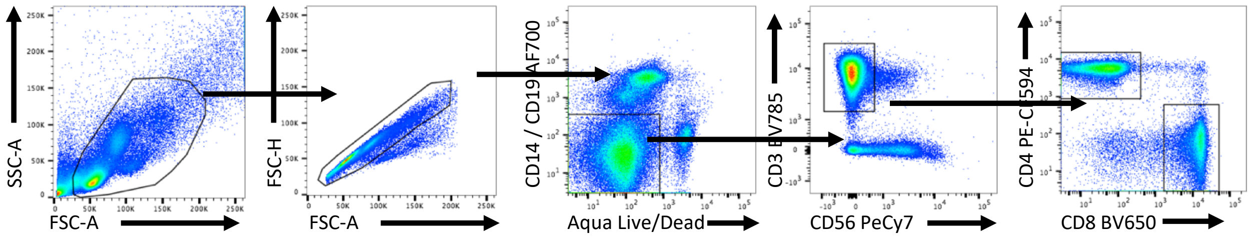

Supplementary Figure 8: Gating scheme for Figure 6 and Supplementary Figure 8

Supplementary Figure 9

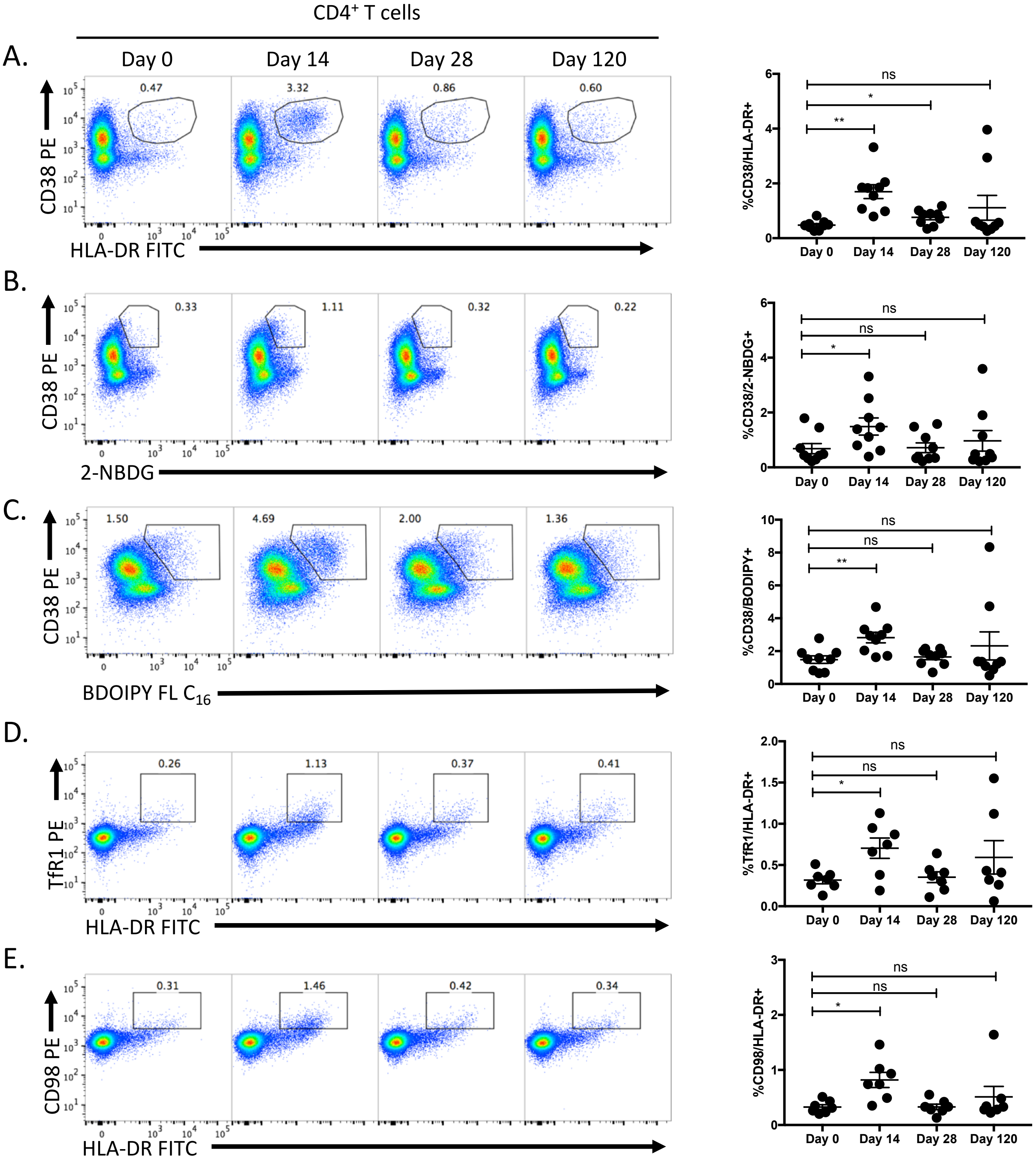

Supplementary Figure 9: Vaccine-reactive CD4⁺ T cells are identifiable by changes in metabolite transporter expression and metabolite utilization. CD4 T cells from TAK-003 recipients were analyzed by flow cytometry at days 0, 14, 28 and 120 post vaccination. Vaccine-reactive CD4⁺ T cells were quantified based on expression of **A)** CD38/HLA-DR, **B)** CD38/2-NBDG, **C)** CD38/BODIPY FL-C16, **D)** TfR1/HLA-DR, or **E)** CD98/HLA-DR. Error bars show mean and SEM. N = 10 individuals. * $p < 0.05$, ** $p < 0.01$ (paired t test). Source data are provided as a Source Data file

Supplementary Figure 10

A.

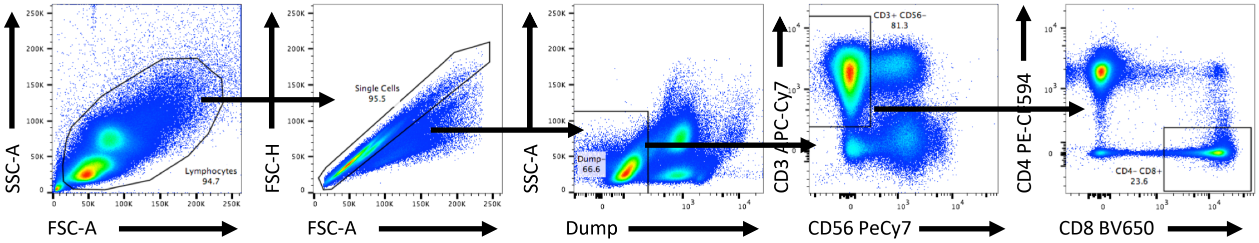

Supplementary Figure 10: Gating strategy for Figure 7. Dump channel contains viability marker (Aqua Live/Dead), CD14 BV510, and CD19 BV510.

Supplementary Figure 11

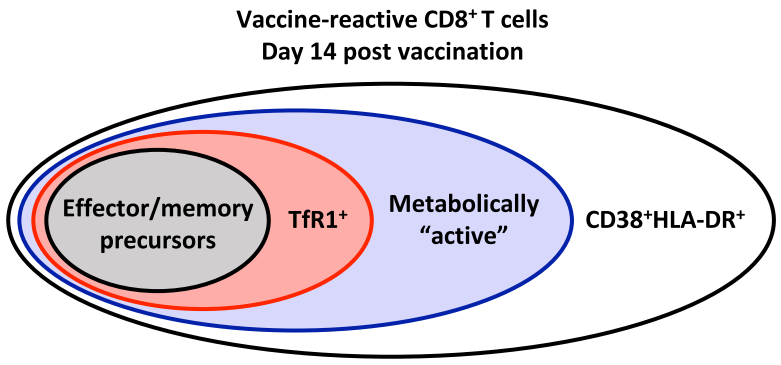

Supplementary Figure 11: Graphical representation of proposed memory precursor model

Supplementary Table 1. Immunodominant epitopes identified by matrix ELISPOT analysis of PBMCs from the individual analyzed in figure 2A

Peptide ID	Length	Sequence	Genomic Location (amino acid #)
NS1-10	18	KQITPELNHILSENEVKL	844 - 861
NS1-20	16	TAECPNTNRAWNSLEV	915 - 930
NS3-30	16	LRTLILAPTRVVAAEM	1,691 - 1,706
NS3-75	16	EGINYADRRWCFDGIK	2,028 – 2,043

Supplementary Table 2. Differential gene expression from scRNAseq analysis of merged CD38^{-}HLA-DR^{-}CD8^{+} T cells and CD38^{+}HLA-DR^{+}CD8^{+} T cells isolated 14 days post TAK-003 administration

Cluster 1				Cluster 2				Cluster 3			
Ensembl ID	Gene Name	p-Value	Log2 FC	Ensembl ID	Gene Name	p-Value	Log2 FC	Ensembl ID	Gene Name	p-Value	Log2 FC
ENSG00000204475	NCR3	1.14E-30	3.10	ENSG00000160789	LMNA	0.0288592	1.20	ENSG00000172005	MAL	1.90E-19	2.91
ENSG00000221869	CEBPD	1.87E-25	2.93	ENSG00000102760	RGCC	0.1811397*	0.98	ENSG00000138795	LEF1	7.83E-11	2.15
ENSG00000256553	TRAV1-2	4.43E-24	2.77	ENSG00000126353	CCR7	0.2861576*	0.97	ENSG00000179144	GIMAP7	4.15E-08	1.82
ENSG00000165272	AQP3	3.71E-21	2.50	ENSG00000109321	AREG	0.2958368*	0.99	ENSG00000265972	TXNIP	2.18E-06	1.60
ENSG00000139187	KLRG1	2.53E-18	2.30	ENSG00000092820	EZR	0.4035891*	0.83	ENSG00000188404	SELL	6.36E-06	1.57
ENSG00000111796	KLRB1	6.44E-14	3.66	ENSG00000130844	ZNF331	0.6379040*	0.75	ENSG00000081059	TCF7	8.65E-06	1.53
ENSG00000140030	GPR65	1.39E-10	1.73	ENSG00000153234	NR4A2	0.8413076*	0.68	ENSG00000100100	PIK3IP1	1.41E-05	1.49
ENSG00000132965	ALOX5AP	8.51E-10	1.66	ENSG00000171223	JUNB	0.9054231*	0.65	ENSG00000131507	NDFIP1	7.73E-05	1.38
ENSG00000100219	XBP1	3.37E-09	1.60	ENSG00000198695	MT-ND6	0.92369497*	0.66	ENSG00000114861	FOXP1	7.80E-05	1.41
ENSG00000107742	SPOCK2	2.98E-07	1.38	ENSG00000177606	JUN	1*	0.63	ENSG00000126353	CCR7	0.0001194	1.44
ENSG00000069667	RORA	1.12E-06	1.35	ENSG00000143384	MCL1	1*	0.49	ENSG00000099204	ABLIM1	0.0008992	1.25
ENSG00000120129	DUSP1	1.61E-06	1.30	ENSG00000177954	RPS27	1*	0.28	ENSG00000132424	PNISR	0.0027191	1.14
ENSG00000113088	GZMK	5.47E-06	1.30	ENSG00000142676	RPL11	1*	0.16	ENSG00000082074	FYB	0.0140815	1.02
ENSG00000168685	IL7R	6.32E-06	1.24	ENSG00000197747	S100A10	1*	0.15	ENSG00000152558	TMEM123	0.0176622	0.98
ENSG00000211747	TRBV20-1	6.53E-06	1.47	ENSG00000122406	RPL5	1*	0.1	ENSG00000127528	KLF2	0.1300802*	0.75
ENSG00000180644	PRF1	6.53E-06	1.31	ENSG00000142937	RPS8	1*	0.09	ENSG00000111678	C12orf57	0.1425691*	0.74
ENSG00000134107	BHLHE40	7.96E-06	1.28	ENSG00000168653	NDUFS5	1*	0.08	ENSG00000111716	LDHB	0.1636998*	0.71
ENSG00000170345	FOS	2.32E-05	1.18	ENSG00000116251	RPL22	1*	0.06	ENSG00000171522	PTGER4	0.1725445*	0.70
ENSG00000151883	PARP8	3.59E-05	1.19	ENSG00000020633	RUNX3	1*	0.04	ENSG00000078596	ITM2A	0.1830545*	0.70
ENSG00000116741	RGS2	4.20E-05	1.21	ENSG00000183508	FAM46C	1*	0.04	ENSG00000133872	SARAF	0.1929737*	0.68

Cluster 4				Cluster 5				Cluster 6			
Ensembl ID	Gene Name	p-Value	Log2 FC	Ensembl ID	Gene Name	p-Value	Log2 FC	Ensembl ID	Gene Name	p-Value	Log2 FC
ENSG00000176890	TYMS	1.28E-45	7.30	ENSG00000126353	CCR7	1.47E-05	1.94	ENSG00000115523	GNLY	6.67E-08	2.45
ENSG00000117632	STMN1	2.07E-35	3.83	ENSG00000172005	MAL	7.41E-05	1.92	ENSG00000137441	FGFBP2	8.69E-06	2.27
ENSG00000196126	HLA-DRB1	2.79E-33	3.69	ENSG00000256039	RP11-291B21.2	0.00229398	1.69	ENSG00000100450	GZMH	3.99E-05	2.09
ENSG00000204287	HLA-DRA	5.97E-32	4.11	ENSG00000138795	LEF1	0.01295731	1.43	ENSG00000100453	GZMB	3.99E-05	2.22
ENSG00000182054	IDH2	8.24E-29	2.98	ENSG00000099204	ABLIM1	0.19874825*	1.02	ENSG00000211714	TRBV7-3	0.00098458	2.76
ENSG00000278030	TRBV7-9	1.49E-23	3.24	ENSG00000196352	CD55	0.21238846*	0.99	ENSG00000275302	CCL4	0.00129037	2.00
ENSG00000222041	LINC00152	2.42E-20	2.57	ENSG00000131507	NDFIP1	0.35696785*	0.87	ENSG00000276070	CCL4L2	0.00193859	2.79
ENSG00000137441	FGFBP2	4.31E-20	2.81	ENSG00000081059	TCF7	0.39989201*	0.87	ENSG00000134539	KLRD1	0.00660648	1.58
ENSG00000100453	GZMB	8.73E-19	2.83	ENSG00000109321	AREG	0.41200657*	0.93	ENSG00000100097	LGALS1	0.01337712	1.64
ENSG00000137309	HMGA1	1.12E-17	2.32	ENSG00000130844	ZNF331	0.48400284*	0.81	ENSG00000223865	HLA-DPB1	0.04209127	1.36
ENSG00000164104	HMGB2	2.79E-14	2.34	ENSG00000111678	C12orf57	0.5365518*	0.76	ENSG00000231389	HLA-DPA1	0.04709035	1.38
ENSG00000019582	CD74	2.79E-14	2.18	ENSG00000114861	FOXP1	0.66101578*	0.73	ENSG00000077984	CST7	0.06235885*	1.28
ENSG00000196230	TUBB	2.79E-14	2.28	ENSG00000112245	PTP4A1	0.66999936*	0.72	ENSG00000105374	NKG7	0.06898583*	1.28
ENSG00000111640	GAPDH	2.79E-14	2.37	ENSG00000163682	RPL9	0.77194459*	0.67	ENSG00000127824	TUBA4A	0.21525376*	1.11
ENSG00000100450	GZMH	2.79E-14	3.00	ENSG00000144713	RPL32	0.78633913*	0.66	ENSG00000197971	MBP	0.38340184*	1.02
ENSG00000103187	COTL1	2.79E-14	2.61	ENSG00000145425	RPS3A	0.78864494*	0.66	ENSG00000150093	ITGB1	0.40835006*	1.05
ENSG00000184009	ACTG1	2.79E-14	2.17	ENSG00000188404	SELL	0.79356275*	0.65	ENSG00000153563	CD8A	0.48414365*	0.95
ENSG00000123416	TUBA1B	2.32E-13	2.07	ENSG00000114942	EEF1B2	0.82617714*	0.64	ENSG00000197540	GZMM	0.50328961*	0.95
ENSG00000198830	HMGN2	3.97E-13	1.96	ENSG00000100100	PIK3IP1	0.82617714*	0.62	ENSG00000197019	SERTAD1	0.54337247*	0.95
ENSG00000231389	HLA-DPA1	1.51E-12	2.05	ENSG00000116251	RPL22	0.83002934*	0.62	ENSG00000172116	CD8B	0.55791841*	0.93

CD38^{+}HLA-DR^{+}				CD38^{-}HLA-DR^{-}			
Ensembl ID	Gene Name	p-Value	Log2 FC	Ensembl ID	Gene Name	p-Value	Log2 FC
ENSG00000204287	HLA-DRA	1.26E-46	6.58	ENSG00000111796	KLRB1	2.45E-20	2.51
ENSG00000196126	HLA-DRB1	3.30E-28	3.63	ENSG00000165272	AQP3	6.55E-07	1.45
ENSG00000117632	STMN1	1.64E-21	3.07	ENSG00000120129	DUSP1	2.15E-06	1.29
ENSG00000103187	COTL1	1.04E-17	2.25	ENSG00000170345	FOS	9.94E-06	1.22
ENSG00000278030	TRBV7-9	1.21E-13	2.49	ENSG00000160789	LMNA	1.52E-05	1.29
ENSG00000100450	GZMH	1.08E-10	1.90	ENSG00000100906	NFKBIA	0.000167779	1.06
ENSG00000164104	HMGB2	1.20E-10	1.76	ENSG00000168685	IL7R	0.000363501	1.02
ENSG00000188404	SELL	9.56E-10	1.66	ENSG00000159388	BTG2	0.000399979	1.02
ENSG00000111640	GAPDH	1.76E-09	1.57	ENSG00000107742	SPOCK2	0.00054224	1.01
ENSG00000019582	CD74	3.17E-09	1.58	ENSG00000099860	GADD45B	0.000577645	1.01
ENSG00000198830	HMGN2	3.83E-09	1.56	ENSG00000110848	CD69	0.000834716	0.98
ENSG00000196230	TUBB	5.63E-09	1.63	ENSG00000172543	CTSW	0.001700994	0.93
ENSG00000137441	FGFBP2	2.81E-08	1.77	ENSG00000144655	CSRNP1	0.001724074	0.94
ENSG00000184009	ACTG1	4.86E-08	1.44	ENSG00000168209	DDIT4	0.002324266	0.92
ENSG00000148908	RGS10	2.53E-07	1.38	ENSG00000152518	ZFP36L2	0.002738483	0.88
ENSG00000123416	TUBA1B	2.75E-06	1.34	ENSG00000198695	MT-ND6	0.003401321	0.93
ENSG00000082074	FYB	1.24E-05	1.20	ENSG00000160888	IER2	0.004893276	0.85
ENSG00000172531	PPP1CA	1.34E-05	1.20	ENSG00000125740	FOSB	0.005352012	0.86
ENSG00000139193	CD27	2.88E-05	1.18	ENSG00000145675	PIK3R1	0.007252764	0.85
ENSG00000090104	RGS1	2.95E-05	1.25	ENSG00000044574	HSPA5	0.007972284	0.83

*Not significant

Supplementary Table 3. Dominant TCR clones from TfR1$^+$HLA-DR$^+$ CD8$^+$ T cells isolated 14 days post TAK-003 administration

TfR1$^+$HLA-DR$^+$ CD8$^+$ T cell clones, day 14 post TAK-003 administration							
	TCRα			TCRβ			
**Frequency**	**CDR3aa**	**V**	**J**	**CDR3aa**	**V**	**D**	**J**
0.1	CAPLGGAGSYQLTF	TRAV21	TRAJ28	CASSPRQGNTGELFF	TRBV7-9	TRBD1	TRBJ2-2
0.075	CAGRGAGSYQLTF	TRAV21	TRAJ28	CASSLLSYEQYF	TRBV7-9		TRBJ2-7
0.0625	CAVRFPRDYKLSF	TRAV21	TRAJ20	CASSPTGTGYYEQYF	TRBV7-9	TRBD1	TRBJ2-7
0.05	CAVRGRGDYKLSF	TRAV1-2	TRAJ20	CASSSAGTLNTGELFF	TRBV7-9	TRBD1	TRBJ2-2
0.0375	CAGAWKNTGFQKLVF	TRAV27	TRAJ8	CASSEWEGNYGYTF	TRBV6-1	TRBD2	TRBJ1-2
0.0375	CALSEAQYNFNKFYF	TRAV19	TRAJ21	CASSIPTSGTLGDTQYF	TRBV5-6	TRBD2	TRBJ2-3
0.0375	CIVRSLINYGQNFVF	TRAV26-1	TRAJ26	CASSSVSYEQYF	TRBV7-9		TRBJ2-7
0.0375	CAVQAGGYSTLTF	TRAV20	TRAJ11	CASAEADNEQFF	TRBV13	TRBD2	TRBJ2-1
0.025	CAVNEAGGFKTIF	TRAV12-2	TRAJ9	CASSLEVFDEQFF	TRBV5-6		TRBJ2-1
0.025	CAESGDSNYQLIW	TRAV5	TRAJ33	CAWSVGGTGELFF	TRBV30	TRBD1	TRBJ2-2

Supplementary Table 4. Differential gene expression from scRNAseq analysis of merged CD38$^+$HLA-DR$^+$CD8$^+$ T cells and TfR1$^+$HLA-DR$^+$CD8$^+$ T cells isolated 14 days post TAK-003 administration

Cluster 1

Ensembl ID	Gene Name	p-Value	Log2 FC
ENSG00000137441	FGFBP2	5.03E-13	2.36
ENSG00000271503	CCL5	9.87E-13	2.09
ENSG00000105374	NKG7	1.51E-08	1.73
ENSG00000100450	GZMH	1.21E-06	1.57
ENSG00000278030	TRBV7-9	6.49E-06	1.65
ENSG00000019582	CD74	7.09E-06	1.40
ENSG00000077984	CST7	8.18E-06	1.42
ENSG00000245532	NEAT1	1.31E-05	1.37
ENSG00000175567	UCP2	2.50E-05	1.31
ENSG00000179344	HLA-DQB1	8.01E-05	1.37
ENSG00000159111	MRPL10	0.00012172	1.25
ENSG00000222041	LINC00152	0.00015146	1.27
ENSG00000139193	CD27	0.00019436	1.21
ENSG00000223865	HLA-DPB1	0.00030964	1.24
ENSG00000204287	HLA-DRA	0.00033473	1.31
ENSG00000275302	CCL4	0.00044781	1.61
ENSG00000134107	BHLHE40	0.00047933	1.21
ENSG00000172116	CD8B	0.00054519	1.17
ENSG00000133321	RARRES3	0.00089478	1.09
ENSG00000150093	ITGB1	0.00135707	1.15

Cluster 2

Ensembl ID	Gene Name	p-Value	Log2 FC
ENSG00000265972	TXNIP	2.47E-13	2.50
ENSG00000172005	MAL	1.83E-12	2.51
ENSG00000100100	PIK3IP1	8.34E-11	2.16
ENSG00000081059	TCF7	3.51E-08	1.91
ENSG00000138795	LEF1	2.37E-07	1.87
ENSG00000179144	GIMAP7	1.30E-06	1.71
ENSG00000131507	NDFIP1	1.68E-05	1.54
ENSG00000169508	GPR183	4.69E-05	1.58
ENSG00000114861	FOXP1	8.54E-05	1.47
ENSG00000251562	MALAT1	0.00054176	1.27
ENSG00000127528	KLF2	0.00069426	1.28
ENSG00000132424	PNISR	0.00096991	1.26
ENSG00000152558	TMEM123	0.00105688	1.26
ENSG00000227507	LTB	0.00119214	1.26
ENSG00000108848	LUC7L3	0.00262805	1.21
ENSG00000245164	LINC00861	0.00366311	1.22
ENSG00000108654	DDX5	0.00647541	1.08
ENSG00000126353	CCR7	0.00903681	1.14
ENSG00000099204	ABLIM1	0.0104046	1.08
ENSG00000105373	GLTSCR2	0.02220148	0.94

Cluster 3

Ensembl ID	Gene Name	p-Value	Log2 FC
ENSG00000126353	CCR7	4.04E-09	2.20
ENSG00000172005	MAL	7.64E-06	1.89
ENSG00000109321	AREG	8.73E-06	2.02
ENSG00000171223	JUNB	0.01049662	1.15
ENSG00000130844	ZNF331	0.01156852	1.17
ENSG00000081059	TCF7	0.02238134	1.11
ENSG00000131507	NDFIP1	0.02289179	1.08
ENSG00000099204	ABLIM1	0.02584505	1.09
ENSG00000227507	LTB	0.03052904	1.07
ENSG00000112245	PTP4A1	0.03529138	1.06
ENSG00000196352	CD55	0.03590761	1.05
ENSG00000100100	PIK3IP1	0.04157423	1.03
ENSG00000138795	LEF1	0.04160725*	1.07
ENSG00000163682	RPL9	0.05916098*	0.94
ENSG00000167552	TUBA1A	0.06067694*	0.96
ENSG00000241343	RPL36A	0.07101436*	0.90
ENSG00000171522	PTGER4	0.0749041*	0.94
ENSG00000059804	SLC2A3	0.08478618*	0.90
ENSG00000105373	GLTSCR2	0.08478618*	0.88
ENSG00000111678	C12orf57	0.08547346*	0.88

*Not significant

Cluster 4

Ensembl ID	Gene Name	p-Value	Log2 FC
ENSG00000111796	KLRB1	3.78E-25	4.05
ENSG00000160789	LMNA	3.09E-13	3.00
ENSG00000124762	CDKN1A	2.30E-10	2.93
ENSG00000165272	AQP3	4.54E-07	2.15
ENSG00000107742	SPOCK2	0.00051623	1.46
ENSG00000120129	DUSP1	0.00051797	1.45
ENSG00000177606	JUN	0.00176111	1.39
ENSG00000168685	IL7R	0.00176111	1.34
ENSG00000160888	IER2	0.00230922	1.31
ENSG00000144655	CSRNP1	0.00341644	1.33
ENSG00000198355	PIM3	0.00630064	1.32
ENSG00000100906	NFKBIA	0.00651548	1.23
ENSG00000092820	EZR	0.00959321	1.20
ENSG00000152518	ZFP36L2	0.01271475	1.16
ENSG00000128016	ZFP36	0.01453321	1.18
ENSG00000168209	DDIT4	0.01509927	1.19
ENSG00000157514	TSC22D3	0.01538907	1.14
ENSG00000170345	FOS	0.02686229	1.12
ENSG00000095794	CREM	0.02762123	1.11
ENSG00000087074	PPP1R15A	0.03000893	1.07

Cluster 5

Ensembl ID	Gene Name	p-Value	Log2 FC
ENSG00000176890	TYMS	1.41E-59	6.73
ENSG00000171848	RRM2	2.37E-47	6.00
ENSG00000117632	STMN1	4.15E-45	4.45
ENSG00000123416	TUBA1B	6.73E-42	4.10
ENSG00000148773	MKI67	3.30E-34	4.42
ENSG00000132646	PCNA	1.48E-31	3.84
ENSG00000166508	MCM7	1.67E-31	3.63
ENSG00000196230	TUBB	7.03E-31	3.36
ENSG00000164104	HMGB2	8.10E-28	3.04
ENSG00000128951	DUT	2.61E-27	3.02
ENSG00000164611	PTTG1	5.65E-25	3.31
ENSG00000164687	FABP5	4.73E-23	2.92
ENSG00000113810	SMC4	6.37E-23	2.98
ENSG00000099901	RANBP1	1.97E-19	2.49
ENSG00000106399	RPA3	8.68E-19	2.45
ENSG00000143401	ANP32E	1.76E-17	2.44
ENSG00000162607	USP1	1.01E-15	2.28
ENSG00000124795	DEK	1.85E-15	2.15
ENSG00000189159	HN1	9.30E-15	2.17
ENSG00000111669	TPI1	1.24E-14	2.08

CD38$^+$HLA-DR$^+$

Ensembl ID	Gene Name	p-Value	Log 2 FC
ENSG00000265972	TXNIP	6.90E-08	2.84
ENSG00000111796	KLRB1	4.94E-06	3.37
ENSG00000171223	JUNB	1.89E-05	2.10
ENSG00000251562	MALAT1	7.96E-05	1.92
ENSG00000227507	LTB	0.00033723	1.87
ENSG00000168685	IL7R	0.00136381	1.71
ENSG00000131507	NDFIP1	0.00269915	1.65
ENSG00000157514	TSC22D3	0.00296655	1.53
ENSG00000127528	KLF2	0.00504828	1.49
ENSG00000152518	ZFP36L2	0.00784242	1.41
ENSG00000105373	GLTSCR2	0.00856073	1.40
ENSG00000130844	ZNF331	0.01138161	1.47
ENSG00000059804	SLC2A3	0.01329687	1.39
ENSG00000177954	RPS27	0.01561848	1.27
ENSG00000168209	DDIT4	0.01611611	1.42
ENSG00000146278	PNRC1	0.01778325	1.28
ENSG00000133639	BTG1	0.01954174	1.26
ENSG00000167552	TUBA1A	0.02537147	1.30
ENSG00000279483	AC090498.1	0.03944363	1.15
ENSG00000213741	RPS29	0.04830312	1.11

TfR1$^+$HLA-DR$^+$

Ensembl ID	Gene Name	p-Value	Log2 FC
ENSG00000164611	PTTG1	4.77E-09	2.30
ENSG00000164687	FABP5	2.85E-08	2.06
ENSG00000117632	STMN1	4.94E-07	1.99
ENSG00000123416	TUBA1B	5.99E-07	1.94
ENSG00000164032	H2AFZ	1.04E-05	1.60
ENSG00000196230	TUBB	1.37E-05	1.70
ENSG00000111639	MRPL51	3.33E-05	1.55
ENSG00000189159	HN1	4.24E-05	1.55
ENSG00000099901	RANBP1	6.81E-05	1.51
ENSG00000111669	TPI1	7.06E-05	1.46
ENSG00000100911	PSME2	8.02E-05	1.45
ENSG00000178741	COX5A	8.69E-05	1.44
ENSG00000106399	RPA3	9.51E-05	1.49
ENSG00000171848	RRM2	0.000184583	1.87
ENSG00000144381	HSPD1	0.000186542	1.42
ENSG00000165672	PRDX3	0.000186865	1.46
ENSG00000126067	PSMB2	0.000192532	1.43
ENSG00000100097	LGALS1	0.000192532	1.68
ENSG00000100567	PSMA3	0.000276036	1.41
ENSG00000164104	HMGB2	0.000305903	1.43

Supplementary table 5. Peptide pools utilized in this study

DENV type	Strain	Protein region	Size (#aa)	Overlap (#aa)	Source	Cat No.
DENV-1	Nauru/West Pac/1974	C/prM	16	11	JPT Peptide Technologies	custom
	Nauru/West Pac/1974	E	13-18	11-12	BEI Resources	NR-9241/ NR-4551
	Singapore/S275/1990	NS1	13-17	11-12	BEI Resources	NR-2751
	Singapore/S275/1990	NS3	14-17	11-12	BEI Resources	NR-2752
	Singapore/S275/1990	NS5	12-17	11-12	BEI Resources	NR-4203
DENV-2	S16803	C/prM	16	11	JPT Peptide Technologies	custom
	New Guinea C	E	15-20	10-11	BEI Resources	NR-507
	New Guinea C	NS1	15-19	10-11	BEI Resources	NR-508
	New Guinea C	NS2a	15-17	11	BEI Resources	NR-2747
	New Guinea C	NS2b	13-17	11-14	BEI Resources	NR-2748
	New Guinea C	NS3	13-19	10	BEI Resources	NR-509
	New Guinea C	NS4a	14-17	11-12	BEI Resources	NR-2749
	New Guinea C	NS4b	12-17	11	BEI Resources	NR-2750
	New Guinea C	NS5	15-17	11-13	BEI Resources	NR-2746
DENV-3	CH53489	C/prM	16	11	JPT Peptide Technologies	custom
	Philippines/H87/1956	E	12-20	10-11	BEI Resources	NR-9228/ NR-511
	Philippines/H87/1956	NS1	13-17	11-12	BEI Resources	NR-2753
	Philippines/H87/1956	NS3	14-17	11-12	BEI Resources	NR-2754
	Philippines/H87/1956	NS5	13-17	11-13	BEI Resources	NR-4204
DENV-4	Singapore/8976/1995	C/prM	16	11	JPT Peptide Technologies	custom
	Singapore/8976/1995	E	12-20	10-11	BEI Resources	NR-9229/ NR-512
	Singapore/8976/1995	NS1	13-17	11-12	BEI Resources	NR-2755
	Singapore/8976/1995	NS3	15-17	11-12	BEI Resources	NR-2756
	Singapore/8976/1995	NS5	13-17	11-14	BEI Resources	NR-4205

Supplementary Table 6. Antibodies utilized for flow cytometry analysis in this study

Antibody	Manufacture	Clone	Cat#	Lot#	Dilution used
CD14 AF700	BD	M5E2	557923	7047605	1:160
CD14 BV510	BD	MOP9	563079	7129954	1:160
CD19 AF700	BD	HIB19	557921	7045883	1:160
CD19 BV510	BD	SJ25C1	562947	6287551	1:160
CD56 PE-Cy7	BD	B159	557747	7319530	1:40
CD98 PE	BD	UM7F8	556077	7299909	1:40
CD107a FITC	BD	H4A3	555800	4042975	1:200
Granzyme B AF700	BD	GB11	560213	7339825	1:20
HLA-DR FITC	BD	G46-6	555811	4342790	1:40
IFN-γ AF647	BD	4S.B3	563495	3221692	1:20
Ki67 AF488	BD	B56	562900	6261744	1:20
CD3 BV785	Biolegend	OKT3	317330	B231963	1:160
CD38 BV421	Biolegend	HIT2	303526	B240476	1:20
CD38 PE	Biolegend	HB-7	356604	B189351	1:40
CD4 PE-Dzz594	Biolegend	RPA-T4	300548	B200505	1:160
CD4 BV605	Biolegend	RPA-T4	300556	B189700	1:160
CD69 APC	Biolegend	FN50	310910	B176306	1:20
CD69 APC-Cy7	Biolegend	FN50	310914	B195699	1:20
CD71 PE	Biolegend	CY1G4	334106	B242701	1:40
CD8 BV650	Biolegend	RPA-T8	301042	B239273	1:320
HLA-DR BV605	Biolegend	L243	307640	B218290	1:80
CD25 APC	Invitrogen	BC96	17-0259-42	4300267	1:20
EOMES eF660	Invitrogen	WD192B	50-4877-42	1988006	1:20
