# Supplementary material for: Dissecting the heterogeneity of DENV vaccine-elicited cellular immunity using single-cell RNA sequencing and metabolic profiling
Source: Nat Commun. 2019 Aug 14;10:3666. doi: 10.1038/s41467-019-11634-7 (PMC6694189; doi:10.1038/s41467-019-11634-7)
Supplement: Supplementary file 3 — Reporting Summary [file 41467_2019_11634_MOESM3_ESM.pdf]

nature research

Corresponding author(s): Adam T. Waickman

Last updated by author(s): 4/1/2019

Reporting Summary

Nature Research wishes to improve the reproducibility of the work that we publish. This form provides structure for consistency and transparency in reporting. For further information on Nature Research policies, see Authors & Referees and the Editorial Policy Checklist .

Statistics

For all statistical analyses, confirm that the following items are present in the figure legend, table legend, main text, or Methods section.

n/a	Confirmed	
☐	☒	The exact sample size (*n*) for each experimental group/condition, given as a discrete number and unit of measurement
☐	☒	A statement on whether measurements were taken from distinct samples or whether the same sample was measured repeatedly
☐	☒	The statistical test(s) used AND whether they are one- or two-sided<br>*Only common tests should be described solely by name; describe more complex techniques in the Methods section.*
☒	☐	A description of all covariates tested
☒	☐	A description of any assumptions or corrections, such as tests of normality and adjustment for multiple comparisons
☐	☒	A full description of the statistical parameters including central tendency (e.g. means) or other basic estimates (e.g. regression coefficient) AND variation (e.g. standard deviation) or associated estimates of uncertainty (e.g. confidence intervals)
☒	☐	For null hypothesis testing, the test statistic (e.g. *F*, *t*, *r*) with confidence intervals, effect sizes, degrees of freedom and *P* value noted<br>*Give P values as exact values whenever suitable.*
☒	☐	For Bayesian analysis, information on the choice of priors and Markov chain Monte Carlo settings
☒	☐	For hierarchical and complex designs, identification of the appropriate level for tests and full reporting of outcomes
☒	☐	Estimates of effect sizes (e.g. Cohen's *d*, Pearson's *r*), indicating how they were calculated

Our web collection on statistics for biologists contains articles on many of the points above.

Software and code

Policy information about availability of computer code

Data collection	BDFACSDiva v6.2 (BD Biosciences), CTL CellCounting v1.5.5.0 (Cellular Technology Limited), CTL ImmunoSpot v7.0.15.2 (Cellular Technology Limited)
Data analysis	FlowJo v10.2 software (Treestar). Loupe Cell Browser (10x Genomics, CA, v2.0.0). Loupe VDJ Browser (10x Genomics, CA, v2.0.0). VDJTools (v1.1.8). Cell Ranger (10x Genomics, CA, v2.1.0). GraphPad Prism 6 Software (GraphPad Software, La Jolla, CA). bcl2fastq2 v2.20 (Illumina)

For manuscripts utilizing custom algorithms or software that are central to the research but not yet described in published literature, software must be made available to editors/reviewers. We strongly encourage code deposition in a community repository (e.g. GitHub). See the Nature Research guidelines for submitting code & software for further information.

Data

Policy information about availability of data

All manuscripts must include a data availability statement. This statement should provide the following information, where applicable:

- Accession codes, unique identifiers, or web links for publicly available datasets
- A list of figures that have associated raw data
- A description of any restrictions on data availability

For all data generated during the course of this project, we will follow the prevailing standards and guidelines in documenting and depositing data sets.

Quality-controlled raw data as well as processed data used in this publications will be made available upon reasonbalbe request. As described in the manuscript, workflows will be exactly described and documented and will allow any external groups to precisely reproduce results from the raw data. Single cell RNA sequencing data will be uploaded to the NCBI Gene Expression Omnibus (GEO) repository.

We will will adhere to the NIH Grants Policy on Sharing of Unique Research Resources including the Sharing of Biomedical Research Resources: Guidelines for

Recipients of NIH Grants and Contracts on Obtaining and Disseminating Biomedical Research Resources.

Field-specific reporting

Please select the one below that is the best fit for your research. If you are not sure, read the appropriate sections before making your selection.

☒ Life sciences ☐ Behavioural & social sciences ☐ Ecological, evolutionary & environmental sciences

For a reference copy of the document with all sections, see nature.com/documents/nr-reporting-summary-flat.pdf

Life sciences study design

All studies must disclose on these points even when the disclosure is negative.

Sample size	No sample size calculations were performed for this study.
Data exclusions	No data were excluded in this analysis
Replication	Other than single cell RNA sequencing experiments, all experiments were repeated at least twice with a minimum of 4 subjects to ensure reproducibility
Randomization	No randomization was applied in this study
Blinding	Investigators were not blinded in this analysis

Reporting for specific materials, systems and methods

We require information from authors about some types of materials, experimental systems and methods used in many studies. Here, indicate whether each material, system or method listed is relevant to your study. If you are not sure if a list item applies to your research, read the appropriate section before selecting a response.

Materials & experimental systems

n/a	Involved in the study	
☐	☒	Antibodies
☒	☐	Eukaryotic cell lines
☒	☐	Palaeontology
☒	☐	Animals and other organisms
☒	☐	Human research participants
☒	☐	Clinical data

Methods

n/a	Involved in the study	
☒	☐	ChIP-seq
☐	☒	Flow cytometry
☒	☐	MRI-based neuroimaging

Antibodies

Antibodies used

All antibodies used for flow cytometry analysis additionally listed in Supplemental Table 6

Antibody Manufacture Clone Cat# Lot#
CD14 AF700 BD M5E2 557923 7047605
CD14 BV510 BD MOP9 563079 7129954
CD19 AF700 BD HIB19 557921 7045883
CD19 BV510 BD SJ25C1 562947 6287551
CD56 PE-Cy7 BD B159 557747 7319530
CD98 PE BD UM7F8 556077 7299909
CD107a FITC BD H4A3 555800 4042975
Granzyme B AF700 BD GB11 560213 7339825
HLA-DR FITC BD G46-6 555811 4342790
IFN-g AF647 BD 4S.B3 563495 3221692
Ki67 AF488 BD B56 562900 6261744
CD3 BV785 Biolegend OKT3 317330 B231963
CD38 BV421 Biolegend HIT2 303526 B240476
CD38 PE Biolegend HB-7 356604 B189351
CD4 PE-Dzz594 Biolegend RPA-T4 300548 B200505
CD4 BV605 Biolegend RPA-T4 300556 B189700

CD69 APC Biolegend FN50 310910 B176306
CD69 APC-Cy7 Biolegend FN50 310914 B195699
CD71 PE Biolegend CY1G4 334106 B242701
CD8 BV650 Biolegend RPA-T8 301042 B239273
HLA-DR BV605 Biolegend L243 307640 B218290
CD25 APC Invitrogen BC96 17-0259-42 4300267
EOMES eF660 Invitrogen WD192B 50-4877-42 1988006

Validation

Antibody specificity was assessed by the manufacturer. All antibodies were titrated prior to use

Flow Cytometry

Plots

Confirm that:

- [x] The axis labels state the marker and fluorochrome used (e.g. CD4-FITC).
- [x] The axis scales are clearly visible. Include numbers along axes only for bottom left plot of group (a 'group' is an analysis of identical markers).
- [x] All plots are contour plots with outliers or pseudocolor plots.
- [x] A numerical value for number of cells or percentage (with statistics) is provided.

Methodology

Sample preparation

Surface staining for flow cytometry analysis was performed in PBS supplemented with 2% FBS at room temperature. Aqua Live/Dead (ThermoFisher, L34957) was used to exclude dead cells in all experiments. Intracellular protein staining was performed using the Foxp3 Fixation/Permeabilization kit (ThermoFisher, 00-5523-00) according to the manufacturer's recommendation.

Instrument

Flow cytometry analysis was performed on a custom-order BD LSRFortessa instrument. Cell sorting was performed on a BD FACSAria Fusion instrument

Software

Flow cytometry data was collected using BDFACSDiva v6.2 (BD Biosciences) and analyzed using FlowJo v10.2 software (Treestar).

Cell population abundance

The post-sort purity of all samples was assessed immediately after sorting. All post-sort purity assessments shown in the relevant figures

Gating strategy

Viable cells were initially defined by FSC/SSC. Single cells were identified by FSC-H/FSC-H. Viable cells were identified by exclusion of cell viability dye. All additional gating strategies are indicated in the corresponding figure or supplemental figure

- [x] Tick this box to confirm that a figure exemplifying the gating strategy is provided in the Supplementary Information.
